# The Promising Future for Complex Innovative Trial Design in Clinical Research

**DOI:** 10.3126/nje.v13i1.53722

**Published:** 2023-03-31

**Authors:** Brijesh Sathian, Edwin van Teijlingen, Indrajit Banerjee, Mohammad Asim, Russell Kabir

**Affiliations:** 1 Geriatrics and long term care department, Rumailah Hospital, Hamad Medical Corporation, Doha, Qatar; 2 Centre for Midwifery, Maternal and Perinatal Health, Bournemouth University, Bournemouth, UK; 3 Sir Seewoosagur Ramgoolam Medical College, Belle Rive, Mauritius; 4 Clinical Research, Trauma and Vascular Surgery, Surgery Department, Hamad General Hospital, Doha, Qatar; 5 School of Allied Health, Anglia Ruskin University, Essex, UK

##  

Clinical research has evolved significantly over the past decades, with new challenges arising that require innovative solutions. The traditional randomized controlled trial (RCT) design has been the gold standard for clinical research for many years, but it may not always be the most appropriate design for addressing complex research questions. Complex Innovative Trial Design (CID) is an emerging approach that integrates novel statistical and methodological techniques to address these difficult challenges.

### The Importance of CID in Clinical Research:

CID offers several potential benefits for clinical research, including the use of novel statistical techniques such as adaptive designs and Bayesian methods that require computer simulation to determine operating characteristics. By leveraging these techniques, CID has the potential to increase the probability of success for clinical trials while simultaneously reducing the resources required for drug development including time and cost [1,2]. Additionally, CID can help to address certain ethical concerns associated with traditional trial designs, such as the use of placebo controls [2].

Adaptive designs, in particular, have emerged as a promising tool for CID. Adaptive designs allow for modifications to the trial protocol during the trial, based on (preliminary) analyses of accumulated data. This allows for greater flexibility in trial design and can lead to more efficient and informative trials [3]. For example, adaptive designs can allow for early stopping of a trial if it becomes clear that the treatment is ineffective, thereby reducing the number of patients exposed to an ineffective treatment.

Another benefit of CID is the potential for increased patient participation in clinical trials. Traditional RCTs often have strict inclusion and exclusion criteria, which can make it difficult for some patients to participate. CID can allow for greater diversity of inclusion criteria resulting in the recruitment of a more diverse patient population, which can improve the generalizability of trial results [4]. Master protocols are comprehensive study protocols that allow for the evaluation of either (a) multiple treatments simultaneously, based on patient-specific characteristics or disease subtypes [4], or (b) more than one drug intervention at the same time [5]. They have emerged as a promising approach to clinical research, providing a more efficient and personalized approach to drug development. By evaluating multiple treatments in a single trial, master protocols can reduce the time and cost required for clinical trials, as well as increase the likelihood of identifying effective treatments. The use of master protocols is particularly useful for diseases with overlapping pathophysiology or treatment targets, where multiple treatments may be effective in different subgroups of patients. Master protocols can also provide personalized treatment options for patients, by tailoring treatment based on patient-specific characteristics, such as molecular or genetic alterations.

Basket trials evaluate the efficacy of a single treatment in multiple diseases with similar molecular features. Umbrella trials, on the other hand, evaluate multiple treatments in a single disease with various molecular characteristics. Platform trials, similar to umbrella trials, evaluate multiple treatments in a single disease, but with the added flexibility of adding or dropping treatments over the course of the trial.

### Challenges of Implementing CID in Clinical Research:

While CID has many potential benefits, there are also significant challenges associated with its implementation. One challenge is the need for specialized expertise in statistical and trial design methods. CID requires a high level of knowledge and experience, which may be a barrier to adoption by some researchers and clinicians.

Another challenge is the lack of regulatory guidance for CID. Regulatory agencies and ethcis committees may be hesitant to approve CID due to a lack of familiarity with this approach. This can result in delays in the approval process, which can be costly and time-consuming.

Finally, there are concerns around the interpretation and communication of CID results. CID often involves more complex statistical methods, which can make it more difficult for non-experts to understand and interpret trial results. It is important to ensure that results are communicated clearly and accurately to all stakeholders, including patients, clinicians, and regulatory agencies.

CID has the potential to revolutionize clinical research by addressing the challenges presented by modern research questions. By integrating novel statistical and methodological techniques, CID can improve the efficiency and effectiveness of clinical trials. While there are challenges associated with CID, these can be overcome with proper education, training, and regulatory guidance. As the demand for more complex clinical trials continues to grow, CID has the potential to become an increasingly important tool for researchers, the pharmaceutical industry, and regulatory agencies.
